# Observations on an Epidemic of Purulent Ophthalmia, Which Occurred during the Autumn and Winter of 1838, in the Children’s Asylum, of the Philadelphia Almshouse, Blockley

**Published:** 1839-08-03

**Authors:** William T. Webb

**Affiliations:** Alabama, Senior Resident Physician


### MEDICAL EXAMINER.

DEVOTED TO MEDICINE, SURGERY, AND THE COLLATERAL SCIENCES.

No. 31.] PHILADELPHIA, SATURDAY, AUGUST 3, 1839. [Vol. II.

*Observations on an Epidemic of Purulent Ophthalmia, which occurred during the autumn and winter of* 1838, *in the Children's Asylum, of the Philadelphia Almshouse, Blockley.* By William T. Webb, M. D., of Alabama, Senior Resident Physician.

[W. W. Gerhard, M. D., Attending Physician.]

The asylum is a large, commodious building, situated at the north-east corner of the almshouse. It would accommodate from one hundred and fifty to two hundred children. At the time of the appearance of this epidemic, the number of children in the asylum was one hundred and twenty. A majority of these were at school during the day, in a large, airy room. The remainder were under the care of nurses in different parts of the building. At night a number of these children slept in the same apartment, and always two in the same bed. Their diet was plain, yet nutritious. The disease first commenced with the children who were kept in the school-room, and was for several days entirely confined to them, but it eventually spread to every part of the building. During its prevalence, but two adults were attacked; both of these cases were comparatively mild. A few days before the commencement of this epidemic, there was a sudden change in the atmosphere; heavy rains and cold weather succeeded to a summer unexampled for heat and drought. Indeed, during the first thirteen days of its prevalence, we had much damp, chilly, disagreeable weather.

After its appearance, we were particularly careful to separate, and prevent, as far as possible, all communication between the diseased and healthy portion of the children. Vetch observes that the "principal cause which gives force and opportunity to the action of contagion, is the crowding individuals together into too limited space:" we therefore not only separated the healthy from those that were affected, but we separated the healthy from each other as much as possible, and also placed as few of those that were diseased in the same apartment, as the nature of things would permit. But, in spite of every precaution used, a majority of all the children were attacked during the epidemic.

Many are the causes which are said to give rise to epidemics of ophthalmia. Beer and Middlemore give as causes—a peculiar state of the atmosphere, the conveyance of the discharge to the eyes of the healthy, sudden and extreme changes in the weather, as to heat or cold, dryness or dampness; long exposure of the eyes to vivid rays of the sun, &c. &c. To these, Scarpa adds disorder of the primæ viæ. Vetch remarks, "Whether the cause of ophthalmia exists in some spontaneous change in the state of the atmosphere, or in the immediate effect of its vitiation by over-crowding individuals togethe , this conclusion is certain, that from a number of persons thus collected together, the constitution is rendered more disposed to be acted on by the exciting cause of disease."

This epidemic first exhibited itself on the first of October, 1838, by the entrance of two children into the infirmary, with the symptoms of ordinary ophthalmia—increased lachrymation, injection of palpebral conjunctiva, swelling and redness of eye-lids, but unaccompanied by the usual symptoms of catarrh. These we at first considered as cases of ordinary conjunctivitis, which had for a long while been prevalent in the asylum. But we were soon convinced, from the severity of the symptoms, and the rapidity with which it spread, that we had an affection of a more serious nature to contend with. On the 2d, eight were admitted; on the 3d, ten; and by the 7th of October, the whole number that entered the infirmary amounted to forty-six. The symptoms during the same period had greatly increased in violence, and presented themselves as follows:

The first symptoms noted after admission were, increased lachrymal secretion, and increased vascularity of the conjunctiva. The sensation of smarting, or pricking, as if sand, or dust, were beneath the palpebræ, we doubt not was present; as the small fasciculi of blood-vessels to which these symptoms are attributed, were constantly met with on the conjunctiva. These were followed by a second set of symptoms, of greater severity. The increased lachrymation gradually diminished, and was succeeded by a mucous secretion, at first thin, but gradually becoming more abundant, more tenacious, and of a yellow straw colour. In some cases this muco-purulent discharge was preceded by a watery secretion, tinged with blood; and again it would be intimately blended with this sero-sanguineous discharge. The first change which occurred in the texture of the eye-lids, was, increased vascularity, with swelling. By degrees they became more enlarged, the cutaneous surface more red; gradually assuming a dark or purple colour. At this period of the disease, the conjunctiva was of a bright scarlet colour, the superficial vessels much enlarged and distended with blood. In many cases we now, likewise, had marked sclerotitis. As the disease advanced, the palpebræ became very much swollen, œdematous, and of a dark leaden or purple colour, which is accurately represented by the engraving of Dr. Vetch, of the external œdema of the palpebræ in purulent ophthalmia. In severe cases, the swelling and œdema of the eye-lids were so great, that we found it impossible to examine the eye for some days. In these cases, when the eye-lid was everted, its whole internal surface seemed to be converted into a fungous substance; the conjunctiva was covered and concealed by fungous vegetations. Whenever we found the internal surface of the palpebræ in this condition, puriform mucus was secreted in great abundance; which, according to Scarpa, is partly secreted by the ciliary glands, but chiefly by the villous and fungous substance into which the internal membrane of the palpebræ and conjunctiva is converted. In these cases also, the intolerance of light begins very early.

*Intolerantia lucis.*—We were not so fortunate, in every instance, as to arrest the disease even in this stage. In many cases, large ulcers formed on the cornea. When this occurred, it was usually between the fifth and ninth days; but we have seen large ulcers formed in twenty-four hours after the commencement of the disease, in spite of the most active treatment which we could adopt.

When convalescence commenced, the swelling and œdema of the palpebræ gradually disappeared,—the conjunctiva lost its scarlet-like tint, and assumed its natural colour. The purulent discharge diminished in quantity, becoming muco-purulent, and finally mucous, and then entirely ceased.

Perhaps we cannot illustrate the nature of this epidemic of ophthalmia better than by subjoining two or three of the many cases which we noted during its prevalence.

Case I.—F. Harmon, æt. 8 years, entered the Infirmary this morning, October 3d, 1838. Both palpebræ red and swollen. Increased lachrymal secretion. Vascularity of conjunctiva greater than natural. Skin hot. Pulse 94, good volume. Bowels constipated. Was directed

℞ Mag. Sulph. ℥ii.

℞ Cuprii Sulph. gr. iii.

Aquæ ℥i. ft. Collyrium.

*4th.* Eye-lids still red, swelling increased, lachrymation still continues, mixed with a muco-purulent secretion. Vascularity and redness of conjunctiva continues; skin less hot and dry; bowels moved twice by the Sulphate of Magnesia. Collyrium continued, with warm fomentations to eye.

*5th.* Redness and swelling not abated in palpebræ; lachrymation ceased, but muco-purulent discharge continues. Conjunctiva is of a light scarlet tint; vessels enlarged and distended with blood; sclerotitis commencing; six American leeches were ordered to each temple; collyrium continued.

*8th.* Palpebræ much swollen, and of a purple colour; conjunctiva as on 5th; sclerotica is of a pink colour, and its vessels much enlarged; the discharge is more tenacious, and resembles pus in appearance; was bled from arm, ℥xii. Emp. Episp. to back of neck. Collyrium Argent. Nit. gr. ii., Aquæ ℥i.

*10th.* Swelling in palpebræ less; conjunctiva less injected with blood; sclerotitis less marked; collyrium continued.

*11th.* Swelling returned in palpebræ; it is of a dark leaden colour; the upper overhangs the lower eye-lid so much that it is impossible to examine the eye; purulent discharge continues; was bled until patient became very pallid; pulse small; collyrium continued.

℞ Hyd. C. Mit. gr. ii.
Pulv. Jalapæ v.—Take at bed-time.

*12th.* Swelling in eye-lids again diminished, but still of a dark purple colour; conjunctivitis and sclerotitis marked; when the palpebræ are everted, their whole internal surface is covered with fungous vegetations; purulent discharge continues abundant; a large ulcer can be seen on cornea of right eye; on left also a small superficial ulcer; ulcers were touched with the nitrate of silver; Tr. Opii dropped in eyes—p. r. n.

*14th.* No change since 12th, except cornea of right eye is more opake, presenting a dark leaden colour; vessels can be seen running directly to ulcer; skin hot and dry; restless during night. Treatment continued; pulv. ipecac. et opii, gr. v. at night.

*30th.* Palpebræ continue swollen and œdematous; still present the purple colour already noted; upper still overhangs lower eye-lid, so much that it is impossible to examine the eye; fungous vegetations still abundant on internal surface of palpebræ. Since fourteenth, leeches have been applied to temples; blister behind each ear; internal surface of palpebræ touched with the argentum nitratum. But little change was apparent from this time until the 30th, during which time the same treatment was continued, paying particular attention to the bowels, &c.; by Nov. 5th, swelling had nearly disappeared from palpebræ; but the purple colour, though much less, still continued; ulcer on right cornea quite large; on left, ulcer still can be seen. Treatment continued; ulcers touched with argent. nit.

This case gradually improved under the treatment which we have stated, when he was discharged, having leucoma of right eye, left perfectly healthy. Discharged Dec. 16, 1830.

Case II.—Edward Sidinger, æt. 9 years, admitted this morning, 11th of October, 1838. Right palpebræ much swollen, red, very painful when touched; a mucous discharge, mingled with a sero-sanguineous fluid, is constantly running from eye; left eye perfectly healthy; was bled, *ad deliquium animi;* quantity of blood taken was ℥xvi.

*12th.* Swelling and redness which the right eye presented on 11th, much diminished; can open eye at pleasure; the discharge has lost its sero-sanguineous character, and has become muco-purulent, much diminished in quantity; left eye remains healthy; conjunctiva of right is of a scarlet colour; vessels enlarged and distended with blood.

℞ Argent. Nit. gr. ii.
Aquæ ℥ii. ft. Collyrium.

*15th.* Slight swelling of palpebræ still continues; slight conjunctivitis; discharge of mucus has not ceased; cornea healthy; treatment continued.

17*th*. No change since 15th worthy of notice; continue treatment.

20*th*. Swelling has disappeared from right palpebræ; some redness continues; conjunctiva more injected than natural; no sclerotitis; discharge nearly ceased; treatment continued.

25*th*. Palpebræ natural; conjunctiva not injected, has assumed its healthy appearance; discharge ceased.

Case III.—Jeremiah Madden, æt. 6 years, robust, healthy child, was sent to the Infirmary to-day, Oct. 5th. Palpebræ swollen, and quite red; conjunctiva very vascular; lachrymation abundant, mixed with a discharge of a mucous character.

℞ Hyd. Chlor. Mit. gr. ij.
Flor. Sulph. v. nocte.

Collyrium.

Liq. Plumb. Subacet. ʒss.

Muc. Medul. Sassaf. ℥i.

Aq. Rosarum q. s. ft. ℥vi.

7th. Palpebræ continue swollen; conjunctivæ are thickened, and very vascular; lachrymation has in a great measure ceased; the discharge of mucus noticed on 5th has assumed a muco-purulent form, and is discharged in great abundance; corneæ clear; collyrium of 5th discontinued, and the following substituted:

℞ Zinc. Sulph. gr. iv.

Aq. ℥i.

℞ Hyd. Chlor. Mit. gr. ij.

Pulv. Jalap v. nocte.

10*th*. There has been a gradual improvement since the 7th; palpebræ less swollen; thickened appearance of conjunctivæ nearly disappeared; vascular appearance of whole eye diminished; purulent discharge continues, and of a straw colour; corneæ continue healthy; treatment continued.

℞ Emp. Epispas. (nuchæ.)

12*th*. Gradual improvement; palpebræ slightly swollen; the scarlet tint of conjunctivæ is disappearing; discharge has again assumed a mucous character; corneæ perfectly healthy; light not painful to eyes; continue treatment.

14*th*. Palpebræ not swollen; conjunctivæ have assumed nearly a healthy appearance; corneæ clear; discharge from eyes nearly ceased; continue treatment.

15*th*. Palpebræ not swollen; conjunctivæ natural; discharge ceased.

*General summary of the treatment pursued.*

Every thing calculated to irritate the eye, if possible, was carefully removed. When the palpebræ were red and swollen, and the conjunctiva of a scarlet colour, attended with increased lachrymation, leeches were applied in number, depending on the severity of the symptoms, followed by some local application. When the discharge became mucous, or muco-purulent, we at the commencement of the epidemic used poultices; but as this secretion was often discharged in great abundance, and the application of poultices prevented its escape from the eye, they were discontinued,—and tepid water and milk were constantly used to keep the eye perfectly cleansed of all irritating matter.

When the disease farther advanced, attended with much inflammatory action, or at the commencement of the disease, where the symptoms were severe, we found bleeding *ad deliquium animi* to be of greater efficacy than any other one remedy. After general or local depletion had been practised, we found the application of blisters to the nape of the neck, behind the ears, or to the temples, of some service. The local applications used varied in their nature and strength, according to the indications to be fulfilled. We gave a trial to most of the collyria recommended in ophthalmia; as a solution of the sulphates of zinc and copper, vin. tr. opii, liq. plumbi acetatis, and a solution of the nitrate of silver. Of these, we found more marked advantage from the nitrate of silver, than any other collyrium. Of this remedy Dr. Vetch speaks in the highest terms. "The slightest application of it, in substance," he remarks, "can often remove the highest degree of morbid sensibility to light, and instantaneously restore quietude to the organ; it can prevent incipient changes, and obviate advanced ones—and may also be used in solution as a valuable sedative."

Mr. Travers remarks that the same observation will apply to acute irritable ulcers. "Upon these," he observes, "a solution of opium often acts as a stimulant, and augments pain, while the nitrate of silver, in solution, as often assuages it."

Strict attention was always paid to the condition of the bowels, keeping them open with purgative or gentle aperient medicines. To prevent the agglutination of the tarsal margins, and to correct the altered state of the meibomian secretion, we used an ointment recommended by Dr. Middlemore; which he directs to be carefully prepared, by rubbing half a drachm of the liq. plumbi acetatis into a half ounce of well-made spermaceti ointment. This was applied two or three times during the day, and at bed time. When the agglutination of the tarsal margins did occur, a mucous or muco-purulent secretion, collected in considerable quantity under the palpebræ, which, when the eyelids were separated, escaped, often blended with a sero-sanguineous fluid, which, when confined, must have been deleterious to the eye, and at the same time caused the patient considerable pain.

Cold applications of various kinds were used at the beginning of the epidemic; without exception, they gave pain, and were soon abandoned. Tepid washes were always more agreeable to the patients, and had a better influence upon the eye; they were used chiefly for purposes of cleanliness, and consisted of simple tepid water, or milk and water. All permanent applications were found so inconvenient to the children, that it was impossible to use them with any effect.

When ulcers appeared on the cornea, if attended with much inflammation, general or local bleeding was practised, according to circumstances; if the ulcer was superficial, a solution of the nitrate of silver was used, in various proportions, from two to ten grains to the ounce. But if the ulcer was circumscribed and deep, or attended with irregular and ragged edges, a saturated solution of the nitrate of silver was applied with a small brush, or it was used in substance. We sometimes found it necessary to administer mercury, in alterative doses. During the acute stage, the diet was farinaceous; it was afterwards varied according to the stage of the disease, and the condition of the patient. The number of cases treated for ophthalmia in the Children's Asylum, and the results, are as follows:—The total number which entered, and which were discharged, was seventy-three. Of these, sixty-two were entirely cured; three had leucoma of both eyes, two, leucoma of one eye; one, a corneal speck on right eye; and five resulted in staphyloma of one eye. In no one case were we so unfortunate as to have a termination in total blindness in both eyes.

When we compare the results of this epidemic with those sometimes met with, we have every reason to congratulate ourselves upon the favourable result. Dr. Gerhard witnessed an epidemic of purulent ophthalmia in the Children's Hospital of Paris, in the summer of 1832, in which a very large proportion of the cases resulted in total blindness. The treatment there pursued was at first purely antiphlogistic, consisting of large applications of leeches, &c. The bad success of this method led to a change; the nitrate of silver was applied to the fungous surface of the palpebræ, in substance, with manifest advantage, and with a very rapid improvement in the results. In our practice, the antiphlogistic treatment was combined with the local application of the caustic, and the advantages of this union were undoubted. Immediate relief always followed the free abstraction of blood, but the disease was not often permanently arrested until the morbid action was modified by the nitrate of silver.

Dr. W. Poyntell Johnston had very extensive opportunities of observing an epidemic of a similar kind, in one of the quarters of Paris. The patients were treated by the celebrated oculist Sichel. The loss of sight was vastly more frequent than in the cases treated at the Children's Asylum. Dr. Johnston assisted us in making the local applications during the greater part of the epidemic, and rendered the most important service.

[Few of our readers can be aware of the disagreeable and fatiguing duties which devolved upon Dr. Webb. The resistance made by the children to the application of remedies to the eyes, rendered his service at once tiresome and difficult. It is an act of simple justice towards him, to state that his unwearying attention to his duties contributed most essentially to the favourable results which were obtained in the treatment of this formidable disease.

